# The impact of Jade Goody's diagnosis and death on the NHS Cervical Screening Programme

**DOI:** 10.1258/jms.2012.012028

**Published:** 2012-06

**Authors:** L Lancucki, P Sasieni, J Patnick, TJ Day, MP Vessey

**Affiliations:** Fulwood House, Old Fulwood Road, Sheffield S10 3TH, UK; HPV and Cervical Cancer Group, Centre for Cancer Prevention, Wolfson Institute of Preventive Medicine, Queen Mary University of London, EC1M 6BQ; NHS Cancer Screening Programmes, Fulwood House, Old Fulwood Road, Sheffield S10 3TH, UK; NHS Cancer Screening Programmes, Fulwood House, Old Fulwood Road, Sheffield S10 3TH, UK; Unit of Health Care Epidemiology, University of Oxford, UK

## Abstract

**Objectives:**

In August 2008 the British reality TV star Jade Goody made public her diagnosis of cervical cancer. In February 2009 it was announced that she was terminally ill and she died a few weeks later. A surge in cervical screening attendances associated with these events was widely reported. This paper aims to quantify the size of that effect across England, its duration, and whether it affected some groups of women more than others.

**Setting:**

The Cervical Screening Programme in England.

**Methods:**

Routinely collected statistics for the months around Jade Goody's diagnosis and death were compared with those for other periods.

**Results:**

About half a million extra cervical screening attendances occurred in England between mid-2008 and mid-2009, the period during which Jade Goody was diagnosed and died; among these were 370 attendances where the test result was suspected neoplasia. At its peak in March 2009, attendance was 70% higher than expected. Increases were seen in both initial and follow-up screening attendances and in colposcopy attendances, and at all ages, though the magnitude was greater for women aged under 50. A substantially greater proportion of the extra attendances of women aged 25–49 on routine recall occurred in women whose attendance was overdue (28% occurred at 60 months or more) and relatively little represented over-screening (8% had been screened within the last 30 months).

**Conclusions:**

The pattern of increased attendance mirrored the pattern of media coverage of Jade Goody's diagnosis and death. It is likely that the increased screening resulted in a number of lives saved.

## INTRODUCTION

Screening behaviour may be affected by many factors. Health services and screening programmes themselves seek to maintain coverage at a high level through regular positive publicity, while recognizing the need at an individual level to provide information on both benefits and risks and to support informed choice. The media can also stimulate behaviour changes by including screening (or diseases screened for) in the storylines of popular shows;^1^ and publicity given to ‘celebrities’ who are diagnosed with such diseases can also lead to changes in behaviour of persons for whom they may be in some way role models.^2^

Jade Goody died of cervical cancer in March 2009, having been first diagnosed in the previous August; she was aged 27. Shortly before her death, when it was announced that she was terminally ill, reports began to appear of a substantial increase in the number of women undergoing cervical screening.^3^ The annual statistics for 2008–09 showed an increase in cervical screening coverage after many years of gradual decline; furthermore, coverage remained at this higher level in 2009–10.^4^ We used data from the cervical screening call-recall database (the ‘Exeter’ system) to assess the magnitude and durability of the ‘Jade Goody’ effect.

## METHODS

The Exeter database holds information about all invitations and cytology tests on women invited for cervical screening. In England, women aged between 25 and 49 are invited every three years and those aged between 50 and 64 every five years. This is a recent change from screening women aged 20–64 ‘at least five-yearly’, which was variously interpreted locally as every three, four or five years. Annual and monthly data about attendances were obtained from 2004 onwards. The information extracted included women's age, region of residence, category of invitation, screening status (i.e. whether they had been previously invited or screened and, if so, how recently) and test result.

The data were extracted using the definitions for creating the tables in the annual statistical bulletin, with one exception. Those women who have screening tests without being prompted by the programme are counted in the bulletins as having been tested outside the screening programme. For this analysis, all women are counted as having been tested within the programme, other than those invited privately or those with no prior recall type (i.e. those not yet called).

The extracts on which this analysis is based were taken in October/November 2010. As the databases are continually being updated the numbers of women whose data were extracted differs slightly from corresponding numbers in the published bulletins; in all years, the numbers in these extracts are the higher – in 2008–09 by about 40,000 attendances, or 1% of the total.

The number of attendances routinely varies from month to month. In order to estimate the excess of tests taken in the months affected by the publicity surrounding Jade Goody's diagnosis and death, Poisson regression models were fitted to the counts for all other months assuming that the numbers of attendances are influenced both by a seasonal effect and a smoothly varying time effect. Different models were fitted to different age-groups and different categories of test (e.g. type of invitation, time since last test, etc.). The seasonal effect was modelled by allowing a separate multiplicative factor for each calendar month and the time effect was modelled in Stata as a natural cubic spline.^5^ Within the model, terms for other factors (e.g. age and invitation type) were also included. The differences between the observed numbers in the months of particular interest and those predicted by the model (applied to all other months) were taken to be the excess in those months. These residuals could either be positive or negative, but on average they are zero for the months on which the models are fitted. It is their values in the months surrounding Jade Goody's diagnosis and death that are taken as the estimated extra number of attendances.

Analysis was performed to examine whether there were variations in the response to the publicity by age, reason for/category of test, time since last test (where expected date of testing varies according to reason for test) and region. In order to estimate the numbers of women tested who might never have returned to the screening programme without the publicity about a celebrity's illness and death, it was assumed that all tests for such women were included among the excess tests; that all the tests occurred at least six months beyond the programme standard for early repeat tests, and beyond 60 months for routine recall; and that the distributions by time since last test for excess tests of all other women were similar to the respective usual underlying distributions by time since last test.

Published data from colposcopy were also examined following anecdotal reports of women who had previously failed to attend for follow up of an abnormal cytology result now reinstating their appointments and attending. These data are not held on the Exeter database. Finally, we analysed press coverage of the events in order to attempt to correlate screening activity with the publicity.

## RESULTS

In the period 2002–03 to 2005–06 there were 4.0 million screening attendances in each year. The number then fell in 2006–07 and 2007–08 to 3.6 million, rose to 4.0 million again in 2008–09 and fell back again to 3.6 million in 2009–10. This very broad analysis suggests that about 0.4 million extra attendances occurred in 2008–09, the year of Jade Goody's diagnosis and death.

**Figure 1:**
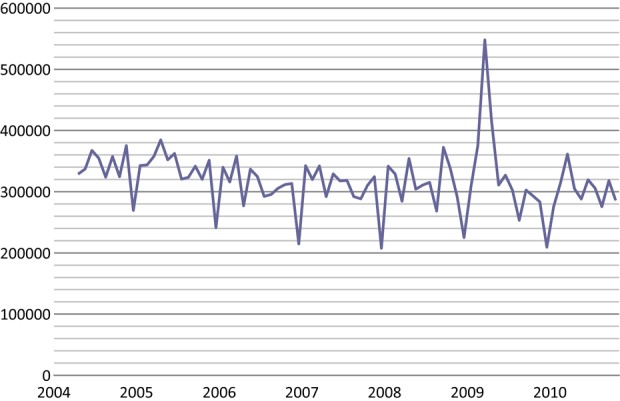
Monthly attendances for cervical screening, England April 2004 to March 2010

The raw monthly data show a more specific timing for these extra attendances when comparing the year of interest with the same months of the previous year (Figure 1). The attendance figure in September 2008 was about 30% above that in September 2007, and October 2008 higher, by about 10%, than October 2007; Jade Goody's diagnosis was made public on 19th August 2008. A far more marked spike in attendances occurred in early 2009. The number of attendances in February 2009 was about 15% higher than in February 2008. Attendances peaked in March at a level just under double that of March 2008 (and 67% greater than expected taking into account other trends); then they fell back to about 15% higher than the corresponding month of the previous year, to about 5% higher in June which could be routine variation and therefore the data from this month have not been included as extra attendances in the analyses. From July onwards, attendance figures were lower in 2009 than in corresponding months of 2008, possibly reflecting a decline due to the changed target age range and recall interval then being implemented in the screening programme or because women who were due for screening in this period had already attended for their tests a few months earlier. These raw monthly data suggest that about 370,000 extra attendances occurred around the time that Jade Goody died; and a further 110,000 or so in the month or two after her diagnosis was made public. Provisional data from the first six months of 2010–11 also suggest a lesser peak in attendances in March 2010 at around the first anniversary of Jade Goody's death.

### Attendances by age

Annual data for 2008–09 show that increases of 15% or more were seen for women aged 25–49, but other age groups showed no or little change (50–54) or even a decline. The number of attendances in age group 20–24 had been dropping swiftly as the raising of the invitation age to 25 was taking effect. The rate of fall in the period of interest was over 15% but a slower decline than had recently been seen. The Poisson model suggests there were 23,000 extra attendances of women not in the target age range, almost all of whom were aged under 25. For the 25–29 age group, adding together the model data from autumn 2008 and spring 2009, there were an estimated 31,000 extra call attendances over five months.

**Figure 2:**
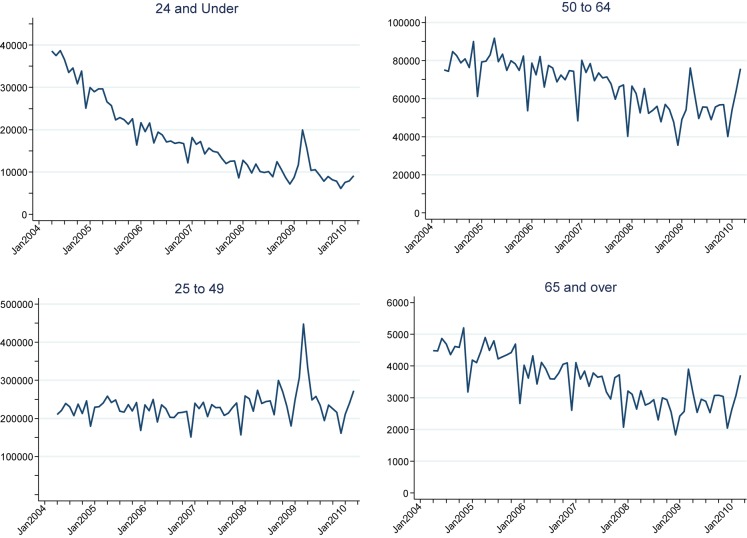
Monthly attendances for cervical screening by age, England April 2004 to March 2010

Large decreases in annual screening rates were seen in age groups 55–59, 60–64 and 65–69 as expected with the standardization of the screening interval to five years. However, Figure 2 shows that the monthly data do indeed show a spike centred on March 2009 across the age groups; and groups below age 50 also show the smaller peak in autumn 2008.

Using the Poisson model to estimate the deviations from trends for each age group separately suggests that overall there were about 105,000 extra attendances in autumn 2008 and 396,000 extra attendances in spring 2009. Of the 501,000 extra attendances, 478,000 were for women in the target age range of 25–64.

### Attendances by invitation category

Of the 478,000 extra attendances of women in the target age range 25–64, 332,000 were for routine recall, 42,000 for first call (mostly aged 25–29) and 65,000 were repeats for surveillance. Repeats following a previous abnormality accounted for 9000 of the extra attendances and repeats following an inadequate sample for 13,000.

### Attendances by time since last test

The Exeter system records, for each attendance, the time since the woman's last test and data were extracted in six-month bands. Distributions by time since last test vary considerably according to the invitation category, so each is considered separately.

About 296,000 of the extra attendances were in women aged 25–49 on routine recall. Of these, 82,000 or 28% occurred at 60 months or more since the previous test, compared with about 17% normally. Conversely, 8% of the extra attendances occurred at under 30 months, compared with about 5% normally. A similar pattern was observed for women aged 50–64.

Repeat attendances for surveillance post colposcopic treatment usually occur about 12 months after a previous attendance. Of the 66,000 extra attendances for surveillance 30,000 (46%) occurred at 18 months or more after the index test; this compares with about 26% normally and suggests that some 18,000 women attended for surveillance after treatment for Cervical Intraepithelial Neoplasia (CIN) who might not otherwise have done so.

Of the 9000 extra repeat attendances following an earlier abnormality, typically required at six months, 2500 (29%) occurred at 12 or more months after the test with the abnormality. Typically, only 17% occur after this length of time, which suggests that some 1200 women were retested who would not otherwise have been.

Repeats following an inadequate sample usually occur less than six months after the inadequate sample; only about 2% take place after 12 months or more. Of the 13,000 extra attendances for a repeat following an inadequate smear, 7000 (55%) were this late. Also it is noteworthy that from summer 2009 onwards the proportion of repeats following an inadequate smear that occurred within six months of the first test was higher than at any time since January 2006.

### Test outcomes

There was an excess of attendances resulting in all test outcomes. The distribution of outcomes in the extra attendances was very similar to the usual distribution of outcomes. Of the results for the 501,000 extra attendances, 6350 were moderate dyskaryosis or worse (1.3%), of which 370 were suspected neoplasia.

### Attendances by region

The increase in attendance showed no major variation by region although there were slight differences in when each region hit its peak.

### Colposcopy appointments

The number of colposcopy appointments rose from 377,000 in 2007–08 to 406,000 in 2008–09 (an 8% increase) and to 454,000 in 2009–10 (a further 12%). There was an increase from 2008–09 onwards in the proportion of appointments where the woman attended – 87% compared with 85% in earlier years (cancelled appointments have been excluded from this calculation).

## DISCUSSION

Previous instances of media cancer stories or celebrity cancer diagnosis have been seen to affect take up of screening.^1,2^ Jade Goody's cervical cancer diagnosis and the publicity prior to and after her death can likewise be documented using screening statistics and confirms the powerful effect a media story or event can have on the public's health-care behaviour.

The extra attendances attributed to the publicity about Jade Goody did not come at a time when the screening programme itself was in a stable state. The changes to the target age range and to the recall interval had been announced in October 2003. However, whilst implementation of the changes had started in spring 2004, a woman's next due date was retained until her next attendance; thus this change to the screening programme had not been fully implemented even by early 2009. This has complicated the analysis and necessitated complex modelling.

When allowances are made for these changes in the structure of the screening programme at around the time of Jade Goody's diagnosis and death, the figures suggest that the extra attendances were from across the whole range of ages in the target age group (plus some outside) and across all types of screening invitation categories. However, the magnitude of the effect appeared to decrease with age; this could be because the women who were closest to Jade Goody in age or circumstances, that is younger women and women with young families, were most affected by her experience.

There was concern that the increased attendance might just have been from the worried well attending for a repeat screen early. This analysis shows that, if anything, the opposite is true – a higher proportion of the extra attendances were among women who were later for their next test than is usual. This was also reflected by the similar proportion of tests with a high-grade result (moderate dyskaryosis or worse) among the extra attendances compared with usual.

As with other reported instances of media-activated increases in screening programme participation, the effect has been broadly limited in time to the duration of the media exposure. The spikes in screening attendances in 2008 and 2009 mirrored the publicity covering initial diagnosis, announcement of terminal illness and death. A study of the extent of magazine coverage in 2008 and 2009 showed a first peak of interest in August and September 2008 and then a much more pronounced peak in March 2009 (26 articles) and April 2009 (19 articles).^6^ Just as the number of magazine articles fell back from May onwards to fewer than 10 per month, so by May (less than three months after the death) cervical screening attendances had settled back at about their previous level.

What was not attempted after the 2001 soap opera and the 2005 Kylie Minogue incidents was a longer term follow-up which could have revealed any lasting effect of the publicity. The spike in attendances following Jade Goody's death was so pronounced that there should be an opportunity to examine the screening programme data in three and five years' time to see whether the initial reactive behaviour translates into a continuing pattern.

Colposcopy information at a national level is too limited to allow much detailed analysis, but the fall in non-attendances may also have contributed to cancer prevention and lives saved. It is not possible to estimate reliably the number of lives saved by this increased screening. In a subsequent analysis, it is intended to examine the morbidity and mortality statistics for any evidence of changes that may be attributed to the increased screening; however, the required data on incidence in 2009 and on mortality in 2011 and 2012 are not yet available.

## CONCLUSION

Jade Goody's diagnosis and death were highly publicized and were marked by a substantial increase in attendances in the cervical screening programme in England. The pattern of increased attendance mirrored the pattern of media coverage. Increases were seen in both initial and follow-up screening attendances and in colposcopy attendances. An increase in screening attendances was observed at all ages, though the magnitude was greater for women aged under 50. It is likely that the increased screening resulted in a number of lives saved but effort should be made to ensure that the extra women reached return for regular screening in the future as the effect of celebrity based publicity appears short lived.
